# Dietary tuna hydrolysate modulates growth performance, immune response, intestinal morphology and resistance to *Streptococcus iniae* in juvenile barramundi, *Lates calcarifer*

**DOI:** 10.1038/s41598-018-34182-4

**Published:** 2018-10-29

**Authors:** Muhammad A. B. Siddik, Janet Howieson, Gavin J. Partridge, Ravi Fotedar, Hosna Gholipourkanani

**Affiliations:** 1 School of Molecular and Life Sciences, Curtin University, 1 Turner Avenue, Bentley, WA 6102 Australia; 2 Australian Centre for Applied Aquaculture Research, Department of Primary Industries and Regional Development, 1 Fleet St, Fremantle, WA 6160 Australia; 3 Centre for Sustainable Aquatic Ecosystems, Harry Butler Institute and School of Veterinary and Life Sciences, Murdoch University, South St, Murdoch, 6150 Australia; 4 Department of Fisheries Science, Faculty of Agriculture and Natural Resources, Gonbad Kavous University, Gonbad - e - Kavous, Iran

## Abstract

This study investigated the effects of tuna hydrolysate (TH) inclusion in fishmeal (FM) based diets on the growth performance, innate immune response, intestinal health and resistance to *Streptococcus iniae* infection in juvenile barramundi, *Lates calcarifer*. Five isonitrogenous and isoenergetic experimental diets were prepared with TH, replacing FM at levels of 0% (control) 5%, 10%, 15% and 20%, and fed fish to apparent satiation three times daily for 8 weeks. The results showed that fish fed diets containing 5% and 10% TH had significantly higher final body weight and specific growth rate than the control. A significant reduction in blood glucose was found in fish fed 10%, 15% and 20% TH compared to those in the control whereas none of the other measured blood and serum indices were influenced by TH inclusion. Histological observation revealed a significant enhancement in goblet cell numbers in distal intestine of fish fed 5 to 10% TH in the diet. Moreover, fish fed 10% TH exhibited the highest resistance against *Streptococcus iniae* infection during a bacterial challenge trial. These findings therefore demonstrate that the replacement of 5 to 10% FM with TH improves growth, immune response, intestinal health and disease resistance in juvenile barramundi.

## Introduction

*Lates calcarifer*, a euryhaline carnivorous fish species commonly known as barramundi or Asian sea bass, is widely cultured throughout the Indo-Pacific region and Australia^1,2^. Currently, the commercial farming of this species is well established in ponds, net cages and recirculating aquaculture systems (RAS) in both fresh and saline water. However, the over-dependence on fishmeal (FM) as a protein source in aqua-diets is regarded as one of the major threats for the sustainable development of aquaculture^3^. Whilst FM is a highly effective protein source in aquatic feeds, issues with supply, increasing prices and environmental concerns are putting pressures on the aquaculture industry to reduce the levels of FM in such diets^4^. Further, mass production and intensive farming of barramundi may possibly result in disease outbreaks^5^ and strategies to mitigate such events are necessary. Hence, suitable alternatives containing bioactive compounds which can both substitute FM and stimulate the defence mechanism of fish are a research priority for a sustainable barramundi industry. Fish protein hydrolysates (FPH) are a possible source of immunostimulants that offer potential growth and immunity benefits against stress and pathogens to the host fish^6,7^.

Fish processing industries produce large volume of by-products, including fins, skin, head, viscera and bones, that are commonly discarded as waste products^8^. These by-products can potentially be used as dietary protein sources in the aquaculture industry following enzymatic hydrolysis, a protein pre-digestion process converting the native proteins into amino acids and peptides suitable for intestinal assimilation. Absorption of peptides through the intestine of vertebrates is a major path of transport and peptides of low molecular-weight are absorbed more rapidly than whole proteins^9^. Recent studies have found that protein hydrolysates with high digestibility, excellent viscosity, good texture, suitable polypeptide fractions and free amino acids, can increase nutrient uptake owing to enhanced biological functionality^10,11^.

The effects of dietary FPH have been evaluated in many commercially important fish species as a partial replacement or supplement to fishmeal^7,12,13^, as immunostimulants to defend against stress and pathogens^7,14,15^ and as attractants to increase diet palatability^16^. The findings of these studies suggest that dietary inclusion of FPH at an appropriate level can have beneficial effects on the feed intake, digestibility, growth performance, innate immunity and specific disease resistance of fish. In addition, most of the studies which have measured innate immune functions have suggested that the immune-reactive peptides in fish protein hydrolysates may play an important role in heightening the innate immunity. The immune-stimulating effects of FPH may therefore result in improved defense against pathogens (phagocytosis and pinocytosis), enhanced lysosomal enzyme activities, enriched alternate complement response, improved hematological defense parameters and enhanced antioxidant activities^14,17,18^.

Presently, several FPHs are effectively used in aqua-feeds for their versatile properties^19–21^ however, no information has been reported on the use of FPH in diets for barramundi. In an effort to diversify the use of FPH in aquaculture, the aim of our study was to investigate whether tuna hydrolysate (TH) is beneficial in terms of growth and immune functions for juvenile barramundi.

## Results

### Growth performance

**Figure 1:**
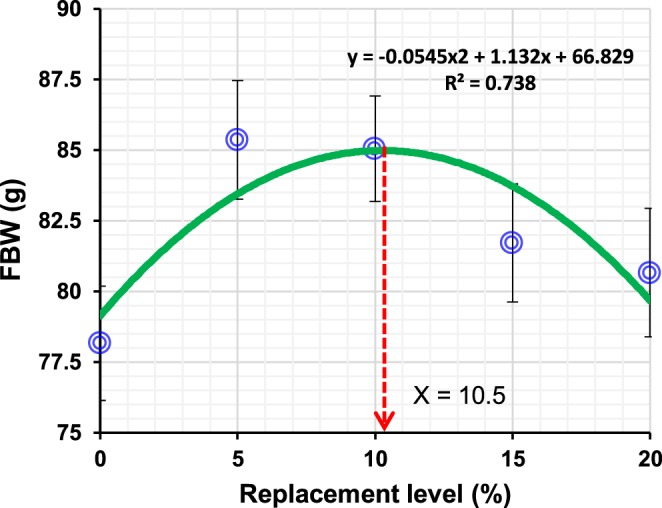
Quadratic regression analysis of final body weight (FBW) for juvenile barramundi fed diets at varying levels of tuna hydrolysate (TH) for 8 weeks. X-axis represents the TH inclusion levels of 0 (control), 5, 10, 15 and 20 are considered as experimental treatments. The multiplication sign ‘X’ represents the TH level for the highest FBW for juvenile barramundi. Each point in the graph represents one treatment with the mean of three replicate groups of fish. The optimal TH level obtained with the quadratic regression analysis for FBW was 10.5% in the diet, respectively.

All tested diets were readily accepted by the juvenile barramundi during the 8 week feeding trial. Growth performance, feed intake (FI), food conversion ratio (FCR) and survival of barramundi fed the four experimental diets and the control are shown in Table 1. Among the dietary groups, significantly greater final body weight (FBW) and specific growth rate (SGR) were observed in the group fed TH05 and TH10 compared to the control, but they were not significantly different from the other treatments. The optimal levels of TH for FBW and SGR were investigated through the quadratic regression analysis (Fig. 1), and the estimated TH inclusion level was 10.5% for the highest FBW. However, the feed utilization indices such as FI and FCR, and survival of fish were not affected by any dietary treatments.

**Table 1:** Growth performance and feed utilization of juvenile barramundi (initial body weight, 12.23 ± 0.41 g) fed tuna hydrolysate (TH) included diets at various levels for 8 weeks.

Parameters	Experimental diets					ANOVA P
	Control	TH05	TH10	TH15	TH20	
FBW (g)	78.17^b^ ± 1.17	85.37^a^ ± 1.79	85.05^a^ ± 1.08	81.72^ab^ ± 1.21	80.67^ab^ ± 1.60	0.021
SGR (%/d)	3.31^b^ ± 0.03	3.47^a^ ± 0.04	3.46^a^ ± 0.02	3.39^ab^ ± 0.03	3.37^ab^ ± 0.04	0.020
FI (g/fish/day)	1.46 ± 0.01	1.47 ± 0.02	1.49 ± 0.01	1.47 ± 0.01	1.46 ± 0.01	0.831
FCR	1.24 ± 0.03	1.13 ± 0.03	1.13 ± 0.03	1.19 ± 0.02	1.20 ± 0.03	0.056
Survival (%)	100.00 ± 0.00	100.00 ± 0.00	98.33 ± 1.67	98.33 ± 1.67	96.67 ± 1.67	0.512

Different superscript letters (a,b,c) in the same row denote significant differences (p < 0.05). Data were represented as mean ± SE.

FBW: mean final body weight (g).

SGR: specific growth rate = [(ln final body weight − ln (pooled initial body weight))/days] × 100

FI: feed intake = dry feed consumed/fish number.

FCR: feed conversion ratio = dry feed fed/wet weight gain.

Survival (%) = (number of final fish − number of initial fish)/number of initial fish × 100.

### Biochemical indices

**Figure 2:**
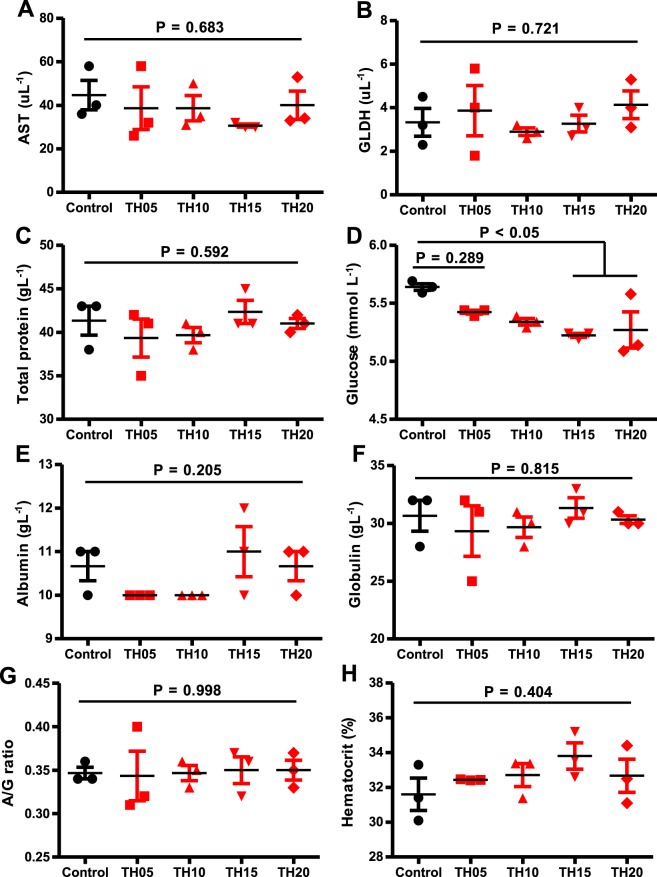
Blood and serum biochemical parameters of juvenile barramundi fed tuna hydrolysate (TH) included diets at various levels for 8 weeks. X-axis represents the TH inclusion levels of 0 (control), 5, 10, 15 and 20 are considered as experimental treatments. (**A**) AST, aspartate transaminase (**B**) GLDH, glutamate dehydrogenase (**C**) total protein (**D**) glucose (**E**) globulin (**F**) albumin (**G**) A/G ratio (albumin/globulin ratio) and (**H**) hematocrit. Data were represented as mean ± S.E., n = 3. Post ANOVA Turkey multiple comparison test was applied to compare the mean value of each treatment with the mean value of the control. Mean values significantly different from the control are noted with P < 0.05.

With the exception of glucose, none of the measured blood and serum biochemical indices including hematocrit, aspartate aminotransferase (AST), glutamate dehydrogenase (GLDH), total protein, albumin, globulin, albumin and globulin ratio (A/G ratio) were influenced by TH inclusion in diets due to large variation between the fish from within the same dietary treatments (Fig. 2). Blood glucose decreased with increasing TH level, with those fish fed TH10, TH15 and TH20 having significantly lower blood glucose than those in the control.

### Histopathology and intestinal morphology

**Figure 3:**
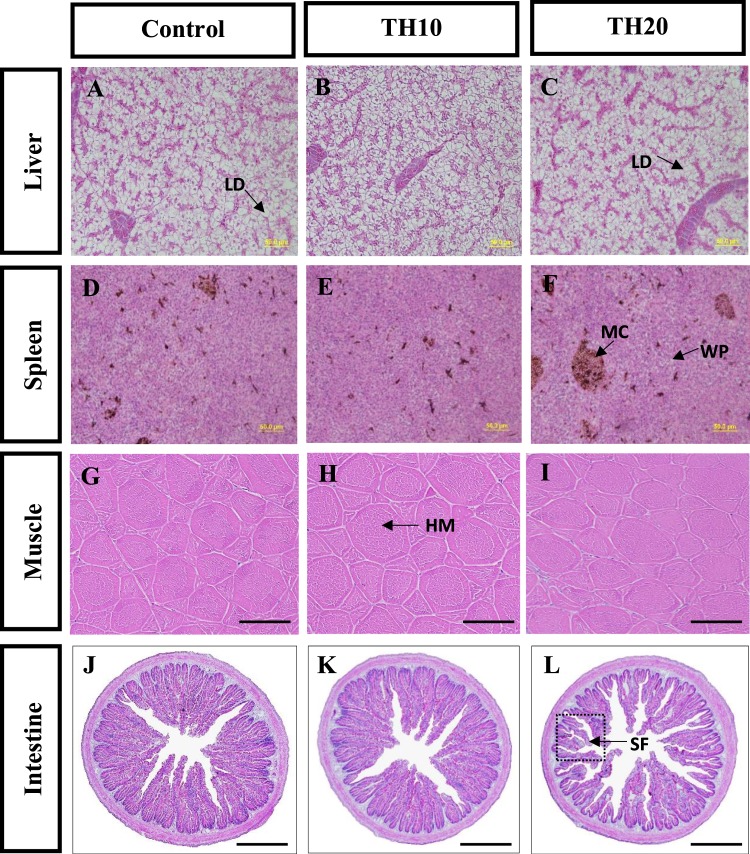
Representative micrographs of liver, spleen, muscle and intestine of juvenile barramundi after 8 weeks of being fed with control, TH10 and TH20. (**A**–**C**) Liver histology from control (**A**) and TH20 (**C**) contain increased lipid deposition in hepatocytes while normal cells were observed in TH10 (**B**) fed fish. (**D**–**F**) Light micrographs of spleen showing marked melanomacrophage aggregates in TH20 (**F**) whereas such cases were not observed in control (**D**) and TH10 (**E**) diets. (**G**–**I**) Muscle tissues containing different diets showed healthy myotomes characterised by rounded, packed and uniformly identical muscle fibres. (**J**–**L**) The distal intestine of fish fed TH20 (**L**) showing reduced mucosal fold lengths and loss of epidermal integrity whereas control (**J**) and TH10 (**K**) fed fish intestinal fold were appear to be healthy with no obvious signs of intestinal inflammation. (LD = lipid droplet; MC = melanomacrophages complex; WP = white pulps; HM = healthy myotome; SF = short fold. All sections are stained with H&E. Scale bar, 50 μm.

Histopathological investigation revealed that those juvenile barramundi fed with TH20 diet had mild to severe alterations in the liver, spleen, and intestinal tissues (Fig. 3). The notable alterations including cytoplasmic vacuolization with an increased amount of lipid accumulation (steatosis) were found in liver of fish fed with TH20 and control diet. However, no histopathological hepatic alterations were observed in fish fed TH10 as indicated by balanced hexagonal hepatocytes with prominent nuclei and rare cytoplasmic vacuolization or granules. Histopathological observation of the spleen revealed higher and bigger melanomacrophage aggregates, increased white pulp and splenic cord in the corpuscles of the spleen of fish fed with TH20 diet than all other diets. The intestinal folds of fish fed TH20 diet were shorter and fewer in number and the lumen was wider, while fish fed all other diets showed histologically normal intestinal folds. No histopathological abnormalities such as muscular dystrophy, injury or necrotic fibres were observed in muscle tissues of fish fed the experimental diets.

**Figure 4:**
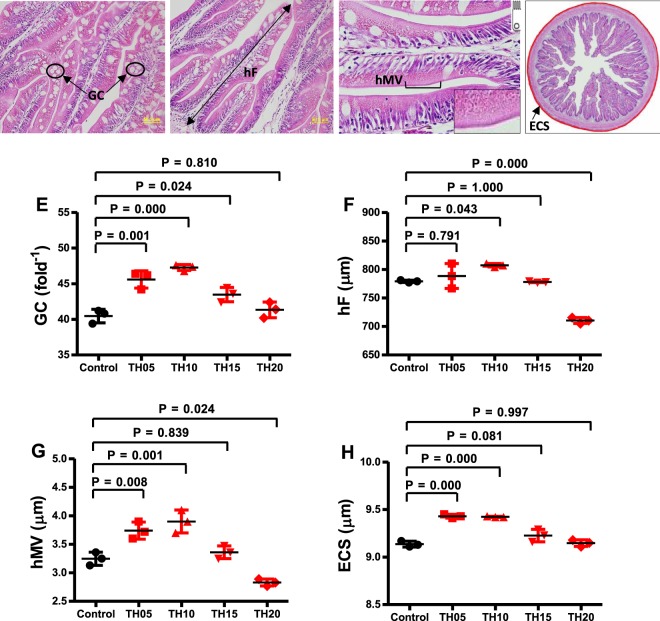
Transverse sections of distal intestine photomicrograph of the juvenile barramundi (Panel A–D). All sections are stained with H&E. Scale bar, 50 μm, inset 20 µm. X-axis represents the TH inclusion levels of 0 (control), 5, 10, 15 and 20 are considered as experimental treatments (**E**–**H**). The distal intestine of juvenile barramundi is influenced by the inclusion of tuna hydrolysate (TH) in diets at varying levels for 8 weeks. The different measurements include GC = Goblet cells (Panel E), hF = fold height (Panel F), hMV = microvillous height (Panel G), ECS = external circumference of serosa (Panel H). Arrow point and cartoon with bracket both indicate hMV (Panel C). Data were represented as mean ± S.E., n = 5. Post ANOVA Tukey multiple comparison test was applied to compare the mean value of each treatment with the mean value of the control. Mean values significantly different from the control are noted with P < 0.05, P < 0.01 and P < 0.001.

The histological measurements of the distal intestine of juvenile barramundi fed diets with different levels of TH are presented in Fig. 4. The micromorphology of intestinal parameters such as goblet cell number per fold (GC), fold height (hF), microvillous height (hMV) and external circumference of serosa (ECS) were altered with the inclusion of TH in diets. The significantly increased GC was found in fish fed 5 to 15% TH included diets whereas increased hMV and ECS were found in TH05 and TH10 diets compared to control. The increased hF was found in fish fed TH05 and TH10 while the decreased hF was observed in TH 20 diet compared to control.

### Lysozyme and complement (ACH50) activity

**Figure 5:**
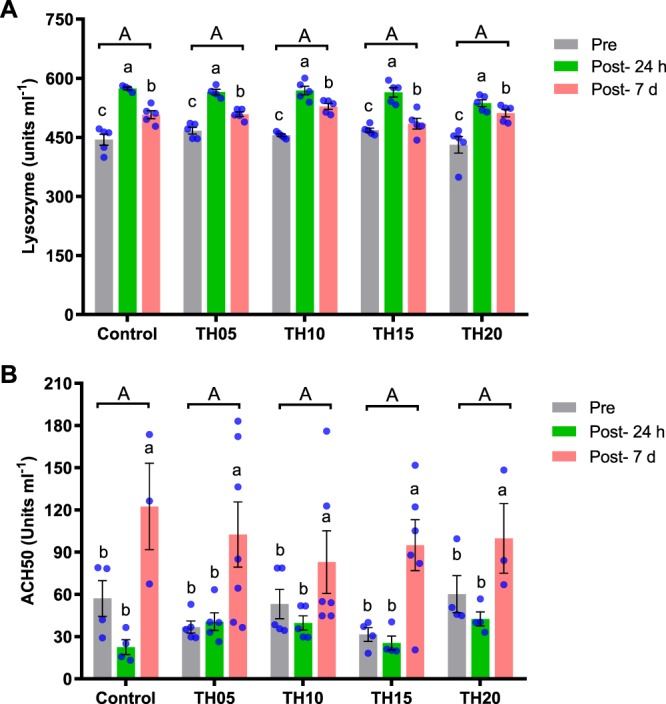
Serum lysozyme (**A**) and complement (**B**) activities of juvenile barramundi fed TH diets at different inclusion levels for 8 weeks. Data were expressed as mean ± SE. X-axis represents the TH inclusion levels of 0 (control), 5, 10, 15 and 20 are considered as experimental treatments. Different lowercase letters (a,b,c) denote statistically significant differences among pre-challenge, post-challenge-24 h and post- challenge- 7 d in the same treatment. Bars holding same uppercase letter (A) among the different experimental treatments are not statistically significant. (Multifactorial ANOVA; Tukey post-hoc test; not significant P > 0.05; significant P < 0.05; P < 0.001).

There was a significant variation observed in the serum lysozyme activities of pre-challenged and post-challenged fish. Fish at 24 hours post-challenge exhibited higher lysozyme activity compared to pre-challenged fish and those 7 days post challenge in all dietary treatments. However, serum lysozyme activity was not influenced by the different inclusion levels of TH in the diets (Fig. 5A). The highest complement activity was registered in fish 7 days post challenge compared to pre-challenge and post-challenge fish at 24 h in all dietary treatments. However, no significant difference was observed between pre-challenge and post-challenge fish at 24 h in all treatments. The complement activity of fish was not influenced by the different inclusion levels of TH in the diets (Fig. 5B). The interactive effects of experimental treatments and sampling period (pre, post-24 and 7 d of challenge) on serum lysozyme activity and complement activity of fish are shown in Table 2.

**Table 2:** Two-way ANOVA analysis on the effect of experimental diets and their challenge period (pre, post-24 and 7 d) and their respective interactions on lysozyme and complement activity.

Parameter	Factors		Interaction
	Challenge period	Diets	Challenge period × Diets
Lysozyme activity	0.000	0.063	0.068
Complement (ACTH) activity	0.000	0.308	0.268

Multifactorial ANOVA; Tukey post-hoc test; not significant P > 0.05; significant P < 0.05; P < 0.001.

### Resistance to infection

**Figure 6:**
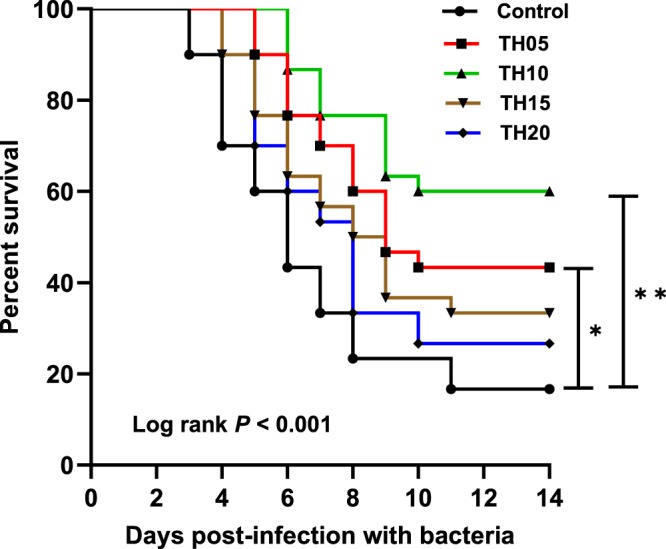
The Kaplan Meyer’s survival analysis of juvenile barramundi after immersion challenge with *Streptococcus iniae*. Survival curves displaying the outcome of bacterial challenge where n = 30 for each treatment. Infection in control started at 3 days post challenge (dpc), 4 dpc in the TH15 and TH20, while infection started in TH05 and TH10 at 5 and 6 dpc, respectively. Significantly higher post challenge survival was found in TH05 and TH10 fish (p = 0.030 and 0.004, respectively) when compared with control. Asterisks * and ** indicate statistically significant difference between treated group and infected control at p < 0.05 and p < 0.01, respectively.

Kaplan-Meier analysis revealed significant differences in survival between treatments (log- rank; χ^2^ (4) = 10.23, p = <0.05). Survival of barramundi following challenge with *S. iniae* was significantly higher in those fish fed TH05 and TH10 diets compared to the control (χ^2^_TH05_ = 4.72, df = 1, p = <0.05 and χ^2^_TH10_ = 8.09, df = 1, p =  < 0.01) whereas dietary groups of TH15 and TH20 exhibited no significant difference compared with the control (χ^2^_TH15_ = 2.74, df = 1, p = 0.098 and χ^2^_TH20_ = 1.25, df = 1, p = 0.263) (Fig. 6).

## Discussion

Fish protein hydrolysates (FPH) derived from raw waste materials produced through enzymatic hydrolysis are regarded as promising aqua-feed ingredients due to their favorable functional^22–24^ and nutritional properties^25,26^. A number of studies have reported that fish hydrolysates are potent growth promoters in fish^27–29^. However, FPH have not been previously studied in barramundi. In this study, tuna hydrolysate (TH) derived from processing by-products was tested in juvenile barramundi and it was found inclusion levels of 5 to 10% enhanced the FBW and SGR. Similar positive growth responses to dietary inclusion of fish hydrolysates have been found in many fish species including olive flounder, *Paralichthys olivaceus*^19^, yellow croaker, *Pseudosciaena crocea*^30^ and Atlantic salmon, *Salmo salar*^29^. The improved growth performance in the present study following moderate levels of hydrolysate inclusion may be a result of the improved availability and subsequent uptake of free amino acids and suitable peptide fractions produced during the enzymatic process which may be beneficial for the growth performance of fish^31^. Amino acids are crucial for a wide variety of protein syntheses with major physiological functions, such as carriers of oxygen, carbon dioxide, vitamins, enzymes and structural proteins^25^. FPH containing free amino acids and suitable peptides has a substantial role in maintaining good health of fish^32^. However, the use of FPH in aqua-feeds must be at the appropriate level as higher inclusion of FPH may negatively influence the growth and feed utilization in fish^1,33^. In Japanese flounder, *Paralichthys olivaceus* 16% or higher inclusion of fish hydrolysate in the diet resulted in significant reduction in growth^34^. Also, an inclusion level of 20% fish hydrolysate in turbot, *Scophthalmus maximus* resulted in significantly reduced specific growth rate (SGR) and feed utilization^31^. In this study, growth performance was significantly elevated at 5 to 10% FM replaced by TH and at further higher replacements (15 to 20%) growth performance started to decline. The detrimental effects of hydrolysates at high inclusion level on fish physiological functioning could be due to an excessive amount of free amino acids (FAA) and peptides of low molecular weight, which may lead to an imbalance in amino acid absorption and saturation of peptide transportation systems^35,36^.

Hematological indices have been considered as valuable biological indicators to assess the health status and physiological condition of fish^37^. The results of the current study showed that dietary inclusion of TH in FM based diets had no significant effect on the hematological indices measured, with the exception of glucose. Likewise, Khosravi *et al*.^19^ found that the addition of protein hydrolysates in low FM diets did not alter most of the hematological indices in juvenile olive flounder, *Paralichthys olivaceus* while some of the health parameters (lysozyme activity, total immunoglobulin) were improved in hydrolysate supplemented groups. In the current study, the concentration of blood glucose was significantly lower in juvenile barramundi fed with 10 to 20% TH included diets compared to those in the control. This result is in accordance with Khosravi *et al*.^13^, who reported the same effect in red sea bream, *Pagrus major* where blood glucose levels were significantly reduced in those fish fed diets containing shrimp hydrolysate. However, another study with the same species found no significant differences in blood glucose levels when fed a diet containing fish hydrolysate^7^. This difference therefore appears to be due to the different types of hydrolysates used between the two studies, but may be due to a number of factors including experimental conditions, fish size and handling methods, as they can strongly affect fish physiological condition^38,39^. The enzymes AST and GLDH are normally measured in fish as the indicators of hepatocellular injury, to determine liver health status. In the present study, the lack of a significant increase in AST and GLDH suggest that the FPH did not cause liver damage). Similarly, Khosravi *et al*.^13^ found no significant difference in serum AST level by the addition of FPH to the diet of red sea bream, *Pagrus major*. However, Cai *et al*.^40^ observed that yellow croaker, *Larimichthys crocea* fed a diet with 40% fish hydrolysates had higher AST levels than fed a control diet.

The intestine, a primary immune organ of the body, *plays* a *major role* in the ingestion and absorption of nutrients, and participates in the protection of the host body through a strong defence against pathogens, allergens and toxins^41^. Some earlier studies have stated that the distal intestine of carnivorous fish is more sensitive in relation to diets and have larger absorptive surface area including villi, microvilli, and higher densities of goblet cells (GC) in the epithelium^42–44^. Furthermore, this part of the intestine has shown the highest variations when alternative protein sources are incorporated in the diets of fish^45^. In the present study, the GC in the intestine were found scattered in order to protect the mucus membranes by secreting mucus^46^. Fish fed TH05, TH10 and TH15 had higher numbers of mucus-secreting GC in the intestine compared to the control. A number of previous studies have reported that GC are positively correlated with the absorption of digestible substances and higher GC results in higher mucosal membrane protection^47,48^. The increment of GC in fish fed the TH05 and TH10 diets might be due to the improved innate immune function against invading microorganisms. These observations are in agreement with an earlier study on red sea bream, *Pagrus major* where dietary inclusion of shrimp hydrolysate in a low fishmeal diet resulted in an increased GC^13^. It is well known that dietary intake of fish has a marked effect on intestinal health, development and function. The longer fold and villus height of intestine are associated with the good health and high absorptive efficiency, whereas shorter fold and villus height are correlated with higher number of pathogenic bacteria in the digestive tract. Moreover, a shortening of the microvillus height can lead to poor nutrient utilization and absorption, reduced immune functions, thereby lower growth performance of fish^49^. According to Dimitroglou *et al*.^50^ good intestinal health in fish is of great importance not only to achieve target growth rates and feed efficiency but also improved the health status of the mucosal epithelium by providing an effective immune barrier against potential intestinal pathogens. In the current study, the histological evaluations in terms of hF, hMV and ECS were increased in fish fed TH05 and TH10 diets might be due to the greater nutrient absorption and utilization results in more surface area for nutrient uptake which was demonstrated by enhanced growth performance of fish. Novriadi *et al*.^51^ reported that the inclusion of 4% squid hydrolysate in the plant based diet partially restore the intestinal inflammation caused by the high inclusion of plant proteins in the diet of Florida pompano, *Trachinotus carolinus*.

When a fish is challenged with pathogens, it is the task of the innate defense system to protect or fight against the pathogens. In order to compensate for a deficiency in the adaptive immune system, fish lysozyme, in the absence of complement has substantial antibacterial activity compared with mammalian lysozymes, not only against Gram-positive bacteria but also against Gram-negative bacteria. Neutrophils and macrophages are the major sources for producing lysozyme^52^. The alternative pathway of complement activity is also an innate component of the immune system protecting fish from invasive pathogens^53^. Multiple studies have suggested that inclusion of FPH in fish diets may stimulate the non-specific immune responses, and this stimulant is strongly influenced by the amount of hydrolysate in the diet^28,54^. However, if the inclusion level of the hydrolysate is too high (>30%), it may have a negative effect in fish^33,54^. The higher lysozyme activity in infected fish demonstrates the defense response to the *S. iniaie* infection in 24 h post challenge and decline at 7 d post-challenge may be explained by granulocyte extravasation from the blood into the peripheral tissues. Interestingly, we observed an opposite pattern in the serum alternative complement pathway activity (ACH50) which had a lower response at 24 h post-challenge compared to 7 d post-challenge. Such an opposite regulation of the immune pathway may indicate that the components of the immune systems in fish species may be regulated in different directions. Since complement acts earlier than lysozyme, which breaks up the resistant layer, lipopolysaccharide, the reduction in alternative pathway may be related to temporary decrease in C3b (cleavage of complement component 3) in the first 24 h post challenge due to usage of this protein in the first hours of response post challenge. On the other hand, according to Ogundele^55^ lysozyme has anti-inflammatory action to inhibit the hemolytic activity of complement, particularly in pathological ranges. Furthermore, a peak response of ACH50 in 7 d post challenge is probably because of a positive feedback loop induced by activation of the classical or lectin pathways^56^. In the current study, fish fed the TH05 and TH10 diets showed higher resistance against infection, while control fish showed the lowest resilience during the 14 days of bacterial challenge. Similarly, dietary administration of FPH increased the disease resistance of various fish, such as red sea bream, *Pagrus major* and juvenile olive flounder, *Paralichthys olivaceus* against *Edwardsiella tarda*^7^ and European sea bass larvae, *Dicentrarchus labrax* to *Vibrio anguillarum*^28^.

In summary, based on the quadratic regression analysis of FBW level, the optimum TH for juvenile barramundi was estimated to be 10.5%. Although the immune parameters (lysozyme, ACTH50) were not affected by TH inclusion in the diets, the increased growth performance and intestinal micro-morphological parameters (GC, hF, hMV and ECS), and decreases in blood glucose level in fish fed TH included diets at moderation might be associated with the improved resistance of juvenile barramundi against *S. iniae* infection, resulting in higher survival during post-challenge. However, further studies on this subject are needed to connecting the linkage between FPH utilization and disease resistance of fish.

## Materials and Methods

### Ethic statements

This study was conducted in strict accordance with the recommendations in the Guide for the Care and Use of Laboratory Animals of Australia. The protocol was approved by the Ethics Committee in Animal Experimentation of the Curtin University (Approval number AEC_2015_41).

### Experimental fish

Following a stringent size grading, a total of 300 healthy juvenile barramundi were sourced from the Australian Centre for Applied Aquaculture Research, Fremantle, Australia and transported to the Curtin Aquatic Research Laboratories (CARL), Bentley, Australia. The fish were then acclimated at CARL for 14 days. During the acclimation period, fish were fed twice a day with a commercially formulated diet (470 g protein kg^−1^ diet and 20.0 MJ kg^−1^ dietary gross energy).

### Experimental diet

All ingredients, except TH were purchased from Specialty Feeds Pty. Ltd, Great Eastern Highway Western Australia. Liquid TH was provided by SAMPI, Port Lincoln, Australia. The dried TH contains 58.4% protein, 1.05% lipid and 11.3% ash. Five isonitrogenous and isocaloric diets were prepared for barramundi having 47% crude protein (CP) and 20 MJ.kg^−1^ gross energy (GE). These diets were labelled as TH0, TH5, TH10, TH15 and TH20 to replace FM at 0%, 5%, 10%, 15% and 20%, respectively by TH. TH0 diet with no replacement was considered as the control. The formulation and proximate composition of the experimental diets are presented in Table 3. The experimental diets were prepared based on the standard method of CARL^57^. All test diets were processed with the addition of water to about 35% mash dry weight of mixed ingredients to form a dough. This dough was then passed through a mincer to create pellets of the desired size (3 mm). The moist pellets were then oven dried at 60 °C for 48 hours and then cooled at room temperature, sealed in plastic bags and stored at −15 °C until further use.

**Table 3:** Formulation and proximate composition of the experimental diets for juvenile barramundi.

Ingredients (g kg^−1^)^a^	Experimental diets				
	Control	TH05	TH10	TH15	TH20
Fish meal	610.0	579.5	549.0	518.5	488.0
Tuna hydrolysate	—	30.5	61.0	91.5	122.0
Wheat	266.0	260.0	254.0	248.0	240.0
Wheat starch	20.0	20.0	20.0	20.0	20.0
Fish Oil	30.0	30.0	30.0	30.0	30.0
Calcium carbonate	2.0	2.0	2.0	2.0	2.0
Salt (NaCl)	2.0	2.0	2.0	2.0	2.0
Vitamin premix^b^	1.0	1.0	1.0	1.0	1.0
Casein	63.0	69.0	75.0	81.0	89.0
Cellulose	6.0	6.0	6.0	6.0	6.0
**Proximate composition (% dry matter)**					
Dry matter	92.72	91.05	90.38	89.71	89.04
Crude protein	47.10	47.16	47.14	47.12	47.18
Crude lipid	9.99	9.88	9.96	9.94	9.92
Ash	13.04	12.56	12.09	11.61	11.14
NFE^c^	22.59	21.45	21.19	21.04	20.80
Gross energy (MJkg^−1^)	19.98	19.97	19.96	19.95	19.97

^a^Supplied by Specialty Feeds, Perth, Australia.

^b^Vitamin premix (g/kg): iron, 10; copper, 1.5; iodine, 0.15;manganese, 9.5; zinc, 25; vitamin A retinol, 100 IU; vitamin D3, 100 IU; vitamin E, 6.25; vitamin K, 1.6; vitamin B1, 1; vitamin B2, 2.5; niacin, 20; vitamin B6, 1.5; calcium, 5.5; biotin, 0.1; folic acid, 0.4; inositol, 60; vitamin B12, 0.002; choline, 150; ethoxyquin, 0.125.

^c^Nitrogen free extracts (NFE) = dry matter − (crude lipid + crude ash + crude protein).

### Experimental conditions and feeding

Following the aforementioned 14 day acclimation period, 300 uniformly sized juvenile barramundi (pool weight of 12.23 ± 0.11 g fish^−1^) were randomly distributed into fifteen independent tanks (300-L water capacity) at a stocking density of 20 fish per tank. Each tank was supplied with constant aeration and water was recirculated from an external bio-filter (Fluval 406, Hagen, Italy) at a rate of 10 L min^−1^. The water quality parameters such as temperature (27.90–29.20 °C), salinity (32–36 ppt), dissolved oxygen (5.92–7.42 mgL^−1^), ammonia nitrogen (<0.50 mgL^−1^) and nitrite (<0.50 mgL^−1^) were monitored daily and were always within the suitable range of fish culture in recirculating aquaculture systems. Fish were kept at 14:10 hr light: dark cycle using *automatic indoor light* switches (Clipsal, Australia). During the experimental period of 8 weeks, fish were fed the treatment diets to satiety three times a day at 0800, 1200 and 1700 h. Fish were starved for 24 h prior to being anaesthetised (AQUI-S^®^, 8 mgL^−1^), weighed and taking blood samples.

### Biochemical indices of blood and serum

At the end of the feeding trial, duplicate blood samples from two anaesthetized fish per tank (six fish per dietary treatment) were withdrawn by caudal vein puncture with a 1 mL non-heparinized syringe. The first set of extracted blood was transferred to heparinised tubes for the determination of haematocrit and blood glucose level. The second set of blood samples were transferred to non-heparinized tubes and allowed to clot overnight. The following day clotted blood samples were centrifuged at 3000 rpm for 15 min at 4 °C, serum was separated and then stored at −80 °C for later measurement of the serum biochemical parameters and immunological indices described below.

Hematocrit (Ht %) was determined by centrifugation of whole blood in glass capillary tubes at 2000 rpm for 5 min following the method of McLeay and Gordon^58^ and expressed as a percentage. A blood glucose meter kit (Accu-Chek, Australia) was used to measure the blood glucose level. Serum biochemical parameters, including aspartate aminotransferase (AST), glutamate dehydrogenase (GLDH), total protein and albumin were measured using an automated blood analyzer (SLIM; SEAC Inc, Florence, Italy) following the methods from Blanc *et al*.^59^. The total globulin content was determined by subtracting the albumin values from the total serum protein values. The albumin and globulin ratio (A/G ratio) was obtained by dividing albumin values by globulin values.

### Histology and intestinal micromorphology analysis

In order to analyze the histopathological condition of liver, spleen, muscle, distal intestine, and histomorphological condition of the intestine, two fish from each replicate were examined (i.e. six juvenile barramundi per dietary treatment) from which blood had previously been extracted. Samples of all tissues were fixed in 10% buffered formalin, dehydrated in ethanol before equilibration in xylene and embedding in paraffin wax. Sections of approximately 5 µm were cut and stained with haematoxylin and eosin (H&E) for histological examination under a light microscope (BX40F4, Olympus, Tokyo, Japan). Digitalized histology images were analyzed using Image J software at different magnification for assessing the height of folds, enterocytes and microvilli according to the procedures described by Escaffre *et al*.^60^ with minor modifications. The number of goblet cells were counted in the highest 10 mucosal folds with the numbers expressed as average number of goblet cells per fold as described by Ramos *et al*.^61^. For gut sample, three cross-sections were quantified for GC, hF, hMV and ECS of the distal intestinal samples.

### Bacterial challenge trial

*S. iniae*, a bacterium pathogenic for barramundi was obtained from the Bacteriology Laboratory, Department of Agriculture & Food, Perth, Australia. The bacteria were grown in trypticase soy broth (Oxoid, Basingstoke, UK) at 24 °C for 24 h and the broth containing the culture was centrifuged at 5000 g for 15 min. The supernatant was discarded and the pellets were washed twice in phosphate-buffered saline (pH 7.2).

At the end of growth trial, 10 average sized fish from each replicate tank were moved to each of 20 × 100 L capacity glass aquaria in separate room in CARL for 14 days bacterial challenge. Of the 20 aquaria, 15 were used for survival assessment counting and 5 were utilised for blood sampling after challenge. The experimental conditions were as follows: water temperature 28.2 °C, salinity 35 g L^−1^, pH 7.6 and photoperiod 14:10 hr light: dark. Following the acclimation, fish were subjected to a bacterial bath challenge with *S. iniae* by removing the fish from the tank and adding them to a bath containing 1.8 × 10^3^ CFU mL^−1^ of the bacteria for 1 minute according to Bromage and Owens^62^. After bathing, fish were returned to their respective aquaria and feeding continued on the treatment diets once per day and fish were closely monitored for bacterial infection. During the challenge period, fish were monitored for signs of infection counted twice daily at 0800 and 1700 h. Infected fish were counted and then removed. Blood sampling for the immune parameters lysozyme and complement activity were conducted before challenge and then again 24 h and 7 d post challenge, and the fish were returned to the respective aquaria after bleeding. To avoid the repeated blood sampling from same fish, fish were tagged individually during stocking.

### Immunological indices of serum

#### Lysozyme activity assay

Serum lysozyme activity was assessed by a turbidimetric assay described by Le *et al*.^63^ with slight modifications. Briefly, *Micrococcus luteus* (0.6 mg mL^−1^) (Sigma) suspension at 0.2 mg mL^−1^ was suspended in sodium phosphate citrate buffer (pH 7.2, 0.05 M) and 30 µL of serum samples were placed into wells of a 96-well plate in triplicate. The mixture was incubated at 25 °C and its absorbance was monitored every 5 min for a total of 30 min at 450 nm with a plate reader. The results are presented as Unit mL^−1^.

#### Alternative complement activity assay

The alternative complement activity was measured using a method modified from Yadav *et al*.^64^ using rabbit red blood cells. Briefly, the rabbit red blood cells (RaRBC) were washed 3 times in 10 mM EGTA-GVB buffer (ethylene glycol tetra-acetic acidmagnesium-gelatin veronal buffer) and then diluted to give 1% suspension containing 2 × 10^8^ cells mL^−1^ in the same buffer. The RaRBC suspension was standardized by adding 100 µL of the 1% suspension to 3.4 mL of distilled water as a blank and the OD of the hemolysate was measured at 405 nm against distilled water to obtain the 100% lysis value. For the blank, red blood cells were similarly mixed with the EGTA –GVB working buffer. A quantity of 100 μL aliquots of serially diluted serum in EGTA –GVB buffer were mixed with 20 μL of red blood cells in a 96 round bottom well plate. The plate was incubated for 90 min at room temperature with gentle shaking every 15 minutes to suspend the RaRBC. After incubation, the plate was centrifuged for 10 min at 800 g at 4 °C. The optical density (OD) of the supernatant was measured at 405 nm using a plate reader. The reciprocal of the serum dilution inducing 50% lysis of RBCs was determined as the ACH50 expressed as unit mL^−1^.

#### Statistical analysis

The data were analysed using SPSS for Windows version 25, IBM Curtin University, Australia. Except lysozyme and complement activity all data was subjected to one-way analysis of variance (ANOVA) followed by Turkey multiple range tests to compare the control diet against each test diet containing tuna hydrolysate (TH). Lysozyme and complement activity were analysed by multifactorial analysis of variance (ANOVA). All results are expressed as means and standard errors (S.E.) with p-values less than 0.05 were considered statistically significant. The FBW was subjected to quadratic regression analysis with TH inclusion levels. The survival graph was constructed using the Kaplan–Meier method and the differences among different dietary groups were performed using log-rank test.

## Data Availability

The datasets generated and analysed during the current study are available from the corresponding author on reasonable request.
